# Molecular genetic analyses of abiotic stress responses during plant reproductive development

**DOI:** 10.1093/jxb/eraa089

**Published:** 2020-02-19

**Authors:** Xinwei Ma, Zhao Su, Hong Ma

**Affiliations:** 1 Department of Biology and the Huck Institutes of the Life Sciences, The Pennsylvania State University, University Park, PA, USA; 2 Trinity College Dublin, Ireland

**Keywords:** Abiotic stresses, large-scale studies, plants, reproductive development, transcriptional regulation

## Abstract

Plant responses to abiotic stresses during vegetative growth have been extensively studied for many years. Daily environmental fluctuations can have dramatic effects on plant vegetative growth at multiple levels, resulting in molecular, cellular, physiological, and morphological changes. Plants are even more sensitive to environmental changes during reproductive stages. However, much less is known about how plants respond to abiotic stresses during reproduction. Fortunately, recent advances in this field have begun to provide clues about these important processes, which promise further understanding and a potential contribution to maximize crop yield under adverse environments. Here we summarize information from several plants, focusing on the possible mechanisms that plants use to cope with different types of abiotic stresses during reproductive development, and present a tentative molecular portrait of plant acclimation during reproductive stages. Additionally, we discuss strategies that plants use to balance between survival and productivity, with some comparison among different plants that have adapted to distinct environments.

## Teaser

This review summarizes the characteristics of plant reproductive development under abiotic stresses, and discusses strategies that plants employ in response to different types of abiotic stresses at reproductive stages.

## Introduction

Plants are sessile and cannot move to avoid unfavorable environmental conditions; therefore, they must cope with adverse environmental conditions by cellular changes. The environmental factors that can limit plant growth and reproduction include water, temperature, light, and nutrients (Zhu, 2016). Fluctuations in these abiotic conditions may lead to minute initial cellular changes and subsequently result in a series of dramatic biochemical, physiological, and morphological changes in plants (Osakabe *et al.*, 2013). These impacts have been extensively studied and discussed for plant responses during vegetative development (Cramer *et al.*, 2011; Bechtold and Field, 2018; He *et al.*, 2018; Kollist *et al.*, 2019). Adverse abiotic factors also negatively impact plant reproduction and yield; such negative effects are responsible for substantial losses in agriculture and economy (Hasanuzzaman *et al.*, 2013; De Storme and Geelen, 2014); therefore, research specifically investigating abiotic effects during reproductive development is of great importance.

Despite the importance, there have been a relatively small number of studies on plant reproductive development under abiotic stresses. This is partly because studies involving reproductive development require longer plant growth periods with more space and greater efforts to obtain the relevant materials. For example, *Arabidopsis thaliana* floral buds are small, requiring meticulous handling of tiny floral organs such as the stamen and pistil (Wellmer *et al.*, 2006; Su *et al.*, 2013). Moreover, the high degree of sensitivity of reproducing plants to environmental changes means that small changes of the growth condition could cause phenotypical differences, making it difficult to recognize phenotypic changes due to gene functional manipulations. More importantly, with the rapid advances of reverse genetics, the lack of obvious phenotypic differences is often considered as no effect due to a mutation, whereas potentially measurable quantitative traits are not investigated, with missed opportunities for uncovering gene functions. Nevertheless, recent advances in technologies such as large-scale data analysis (Su *et al.*, 2013; Ma *et al.*, 2014; Großkinsky *et al.*, 2018) have provided powerful tools to further explore plant response to stresses in reproductive tissues at the molecular level. Here, we summarize studies (Table 1) that explored plant reproductive development under abiotic stresses, and discuss strategies that plants employ in response to different types of abiotic stresses at reproductive stages.

**Table 1.**
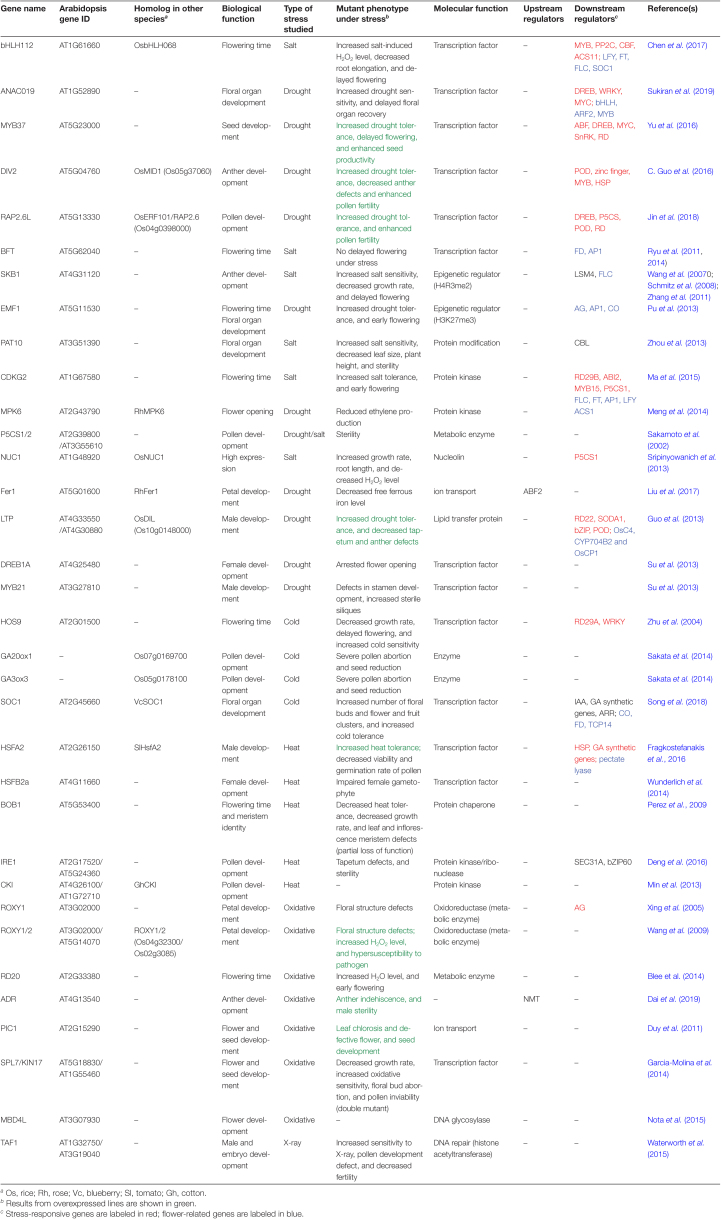
List of genes involved in plant abiotic stress response

## Water deficiency and salinity

**Fig. 1.**
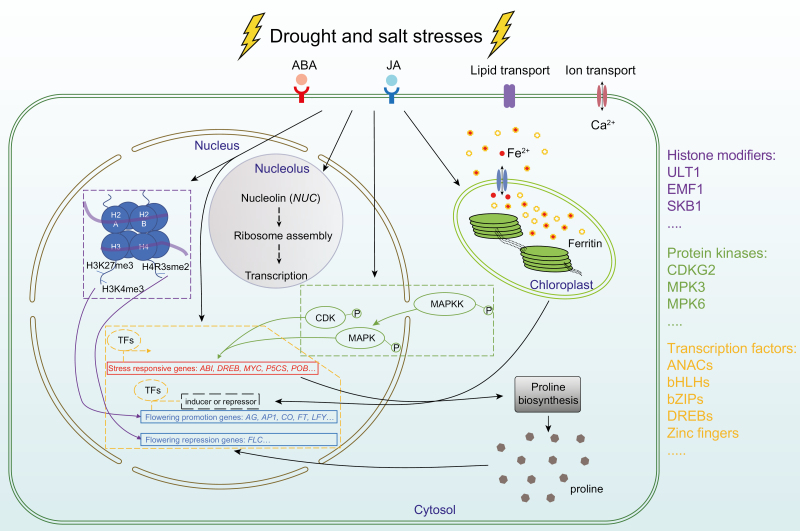
Cellular responses to drought/salt stresses during reproductive development. Transcriptional regulation, epigenetic regulation, and protein modification are labeled in yellow, purple, and green boxes, respectively. Key regulators discussed in this review are listed with the same color codes.

Water availability is one of the most important abiotic factors that affect plant growth and development. Both drought and salt stresses can lead to water scarcity, especially given that both stresses change osmotic homeostasis, accumulate similar metabolites, and can activate overlapping signaling pathways (Zhu, 2002, 2016). Drought is known to cause flowering time change, flower abortion, reduction of pollen fertility and seed number, as well as immature or aborted seeds in plants (Su *et al.*, 2013; Shavrukov *et al.*, 2017). Salt stress also has similar negative effects on reproductive development in rice, wheat, and grape (Khan and Abdullah, 2003; Zheng *et al.*, 2010; Baby *et al.*, 2016). Notably, drought/salt stress also cause differential expression of various genes, including those regulating flowering time and development of reproductive organs, and genes encoding transcription factors (TFs) that function in response to osmotic stresses (Su *et al.*, 2013). Below, we discuss molecular genetic analyses of gene functions and genes identified using large-scale experiments (Fig. 1).

### Transcriptional regulation

Drought/salt stresses impact several aspects of plant reproductive development at the molecular level, especially via transcriptional regulation. Members of several TF families have been shown to respond to water stress and were recently found to play crucial roles in plant reproductive development, including ANAC (abscisic acid-responsive NAC), bHLH (basic helix–loop–helix), DREB (dehydration responsive-element binding), homeobox domain, MYB, and zinc finger, in both *A. thaliana* (Krishnaswamy *et al.*, 2011; Ryu *et al.*, 2011, 2014; Hichri *et al.*, 2014; Yu *et al.*, 2016; Chen *et al.*, 2017; Sukiran *et al.*, 2019) and rice (*Oryza sativa*) (C. Guo *et al.*, 2016; Jin *et al.*, 2018; Minh-Thu *et al.*, 2018).

In general, changes in floral morphology, seed production, and related gene expression patterns together support the importance of the gene function in reproductive development under stress. In an Arabidopsis study in which plants were treated with drought starting at the onset of flowering to the end of their life cycle (Su *et al.*, 2013), the drought stress caused an arrest of the development of floral buds. However, plants recovered partially after acclimation over a period of ~2 weeks of treatment, with reduced seed numbers compared with well-watered plants. Floral defects were observed at different flower stages in drought-treated plants, including abnormal anther development, lower pollen viability, reduced filament elongation, ovule abortion, and failure of the flower to open.

Among genes induced by drought stress during plant reproductive development, Arabidopsis *ANAC019* (Sukiran *et al.*, 2019) and *AtMYB37* (Yu *et al.*, 2016), and rice *OsMID1* (MYB-like family protein) (C. Guo *et al.*, 2016) and *OsERF101* (encoding an AP2 family TF) (Jin *et al.*, 2018) are highly expressed in reproductive organs under drought. These genes can mitigate anther defects and improve pollen fertility and seed production under drought stress. In addition, gene expression analyses in mutants or overexpression lines for these TFs have revealed that important regulatory genes were involved in response to drought during reproductive development, including abscisic acid (ABA) insensitive (*ABI*), *DREB*, *MYB*, peroxidase (*POD*), and responsive to dehydration (*RD*) genes.

**Fig. 2.**
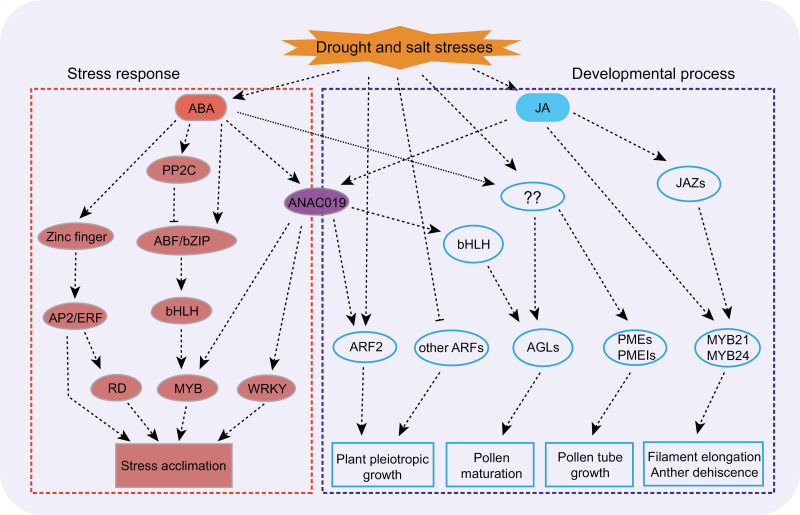
A transcriptional regulation network for flowering under drought/salt stresses, modified from: Su Z, Ma X, Guo H, Sukiran NL, Guo B, Assmann SM, Ma H. 2013. Flower development under drought stress: morphological and transcriptomic analyses reveal acute responses and long-term acclimation in *Arabidopsis*. The Plant Cell 25, 3785–3807, (www.plantcell.org), ‘Copyright American Society of Plant Biologists’. Ovals represent genes with known functions in either stress response or developmental process; boxes show different proposed functions. Dashed lines with arrows represent positive regulatory relationships, whereas hammer-ended dashed lines represent inhibitions. Red symbols indicate stress-related interactions, whereas cyan symbols indicate developmental regulation.

Specifically, *ANAC019* functions as an early drought response regulator, which can be induced in flowers soon (3 d) after drought treatment (Su *et al.*, 2013). The mRNA level of *ANAC019* is much higher in the inflorescence than in the leaf when plants are grown with insufficient water, especially when plants recover after a period (7–14 d) of acclimation to severe drought (Sukiran *et al.*, 2019). The *anac019* mutant showed significant shortening of the stamen and pistil, and delayed recovery of flowering under drought stress as compared with the wild-type (WT) plant. In addition, several important drought-responsive and floral genes were expressed differentially in the *anac019* mutant compared with the WT, consistent with mutant phenotypes. These observations support a critical role for *ANAC019* in promoting not only reproductive development but also drought tolerance (Fig. 2) (Sukiran *et al.*, 2019). Another Arabidopsis gene, *AtMYB37*, also plays an important role in drought response and regulation of seed production, as overexpression lines displayed both increased seed production and drought tolerance; moreover, the expression levels of several key ABA-responsive genes were altered in these lines (Yu *et al.*, 2016), supporting the idea that AtMYB37 affects reproductive development under drought at least in part via transcriptional regulation of key target genes.

In rice, *OsMID1* (C. Guo *et al.*, 2016), encoding a MYB-like TF, is expressed in vascular tissues, can be induced by drought in rice flowers, and its overexpression resulted in enhanced drought tolerance, with reduced anther defects and increased pollen fertility and grain production. A ChIP experiment demonstrated that OsMID1 binds to the promoter region of both drought-responsive (*OsHSP17.0* and *OsCYP707A5*) and anther developmental (*OsKAR*) genes, further supporting its roles in both processes (C. Guo *et al.*, 2016). Another rice gene, *OsERF101*, is also preferentially expressed in flowers, and transgenic lines with overexpression of *OsERF101* displayed elevated peroxidase activity and proline content, which in turn contributed to increased drought tolerance and improved pollen fertility. Additionally, these transgenic plants showed induced expression of ABA-responsive genes, including *RD22*, *LEA3*, and *POD* (Jin *et al.*, 2018). Together, these Arabidopsis and rice results strongly support the roles of TFs in drought response during reproductive development.

However, the functions of some regulators in drought response and development are not similarly conserved. For example, members of the large bHLH family, *AtbHLH112* in Arabidopsis and *OsbHLH068* in rice, function similarly in drought/salt stress response (Chen *et al.*, 2017), but act in opposite manners in Arabidopsis flowering control. It was demonstrated that the *Atbhlh112* mutant exhibits reduced salt tolerance, whereas the overexpression of *OsbHLH068* in Arabidopsis decreases levels of the reactive oxygen species (ROS) H_2_O_2_ and enhances salt tolerance. However, the *Atbhlh112* mutant and *OsbHLH068* overexpression lines both exhibit late-flowering phenotypes. Furthermore, similar expression patterns of stress-responsive genes were found in both *OsbHLH068*- and *AtbHLH112*-overexpressing Arabidopsis lines, whereas the key flowering control gene *FLOWERING LOCUS T* (*FT*) shows the same pattern in an *OsbHLH068*-overexpressing Arabidopsis line and the *Atbhlh112* mutant line. It is possible that the function of these two *bHLH* genes in stress response was ancestral, but the Arabidopsis and rice genes have diverged functionally in control of flowering time.

Although the molecular mechanisms are still unclear for regulation of downstream genes by the above-mentioned TFs, some studies have begun to address this question. It is known that salt stresses can delay flowering in Arabidopsis (Riboni *et al.*, 2014), but a loss-of-function mutant of the *AtBFT* gene, encoding an FT/TERMINAL FLOWER 1 (TFL1) family protein, fails to delay flowering under salt stress (Ryu *et al.*, 2011). The expression of a key regulator of flower development, *AP1*, is reduced in salt-stressed plants, but the repression of *AP1* expression is less severe in the *atbft* mutant than that in the WT (Ryu *et al.*, 2011). Therefore, *AtBFT* functions as a floral repressor under high salinity. Furthermore, under high salinity, AtBFT interferes with the interaction between FT and FD, which are positive regulators of flowering (Ryu *et al.*, 2014). AtBFT accomplishes this interference by binding to the C-terminal region of FD (a bZIP protein that associates with FT), the same region as for FD–FT interaction. Thus, the binding of FD by AtBFT under high salinity results in a reduced level of the FD–FT complex, thus resulting in delayed flowering. This study indicates that specific TFs that are induced under drought/salt stress can be linked to existing regulatory pathways for flower development. Delayed flowering is usually caused by the repression of flowering time genes. This strategy allows plant to conserve energy and resources to survive the unfavorable environment. Future studies may yet reveal other mechanisms for transcriptional regulation.

### Epigenetic regulation

Another level of the regulation of gene expression is epigenetic regulation, which can impact seed germination, phase transition, flowering time control, vegetative and reproductive development, as well as defense and stress response (Chinnusamy and Zhu, 2009; Pikaard and Mittelsten Scheid, 2014). One important and widely investigated aspect of epigenetic regulation is histone modification, which is involved in flowering and salt stress response (Zhang *et al.*, 2011; Pu *et al.*, 2013). One type of histone modification is methylation on an arginine (R3) residue of the subunit histone 4 (H4) and is carried out by arginine methyltranserases (Wang *et al.*, 2001). The Arabidopsis gene (*AtSKB1*) encoding a protein arginine methyltransferase negatively regulates the expression of a key flowering repressor gene *FLOWERING LOCUS C* (*FLC*) through the methylation of H4R3 to yield H4R3sme2 (histone4 arginine3 symmetric dimethylation2), with a consistent late-flowering phenotype in the *atskb1* mutant (Wang *et al.*, 2007; Schmitz *et al.*, 2008). Furthermore, the *atskb1* mutant is also hypersensitive to salt stress. Salt stress can cause dissociation of the AtSKB1 protein from the chromatin, resulting in a reduced level of H4R3sme2 and increased expression of both *AtFLC* and stress-responsive genes (Zhang *et al.*, 2011). It was also found that salt stress caused an increased methylation of the small nuclear ribonucleoprotein Sm-like4 (AtLSM4, related to mRNA splicing) and the *atlsm4* mutant is sensitive to salt, suggesting that regulation of mRNA splicing might be important in tolerance to salt. The authors therefore proposed that, under salt stress, plants exhibit decreased H4R3sme2 and altered splicing, inducing stress-responsive and floral repressor genes, and thus achieving salt tolerance and delayed flowering.

Another example of epigenetic regulation of flowering under drought involves the *EMBRYONIC FLOWER1* (*AtEMF1*) gene, which is important for Polycomb group-mediated transcriptional repression of floral MADS box genes (Pu *et al.*, 2013). In general, Polycomb group proteins act in an opposite fashion to the Trithorax group factors in regulating gene expression (de la Paz Sanchez *et al.*, 2015; Schuettengruber *et al.*, 2017). Pu *et al.* (2013) found that *AtEMF1* is required for histone 3 lysine 27 trimethylation (H3K27me3), whereas the *ULTRAPETALA1* (*AtULT1*) gene encoding a Trithorax group factor is a positive regulator of histone 3 lysine 4 trimethylation (H3K4me3). The functions of AtEMF1 and AtULT1 are antagonistic in regulating the expression of target genes, including *AGAMOUS* (*AG*), *APETALA1* (*AP1*), *FLC*, and *CONSTANS* (*CO*). It was also found that reducing the *AtEMS1* function increased salt tolerance and expression of salt-responsive genes, whereas such effects of reducing AtEMS1 function were cancelled by a mutation in *AtULT1*. Therefore, the *AtEMF1* and *AtULT1* system controls the homeostasis of histone mark deposition and enables proper flowering under salt stress.

### Protein phosphorylation and palmitoylation

Protein modifications, particularly protein phosphorylation, are important for multiple cellular processes, such as cell cycle progression controlled by cyclin-dependent kinases (CDKs), and intracellular signaling regulated by the mitogen-activated protein kinase (MAPK) cascade (Rodriguez *et al.*, 2010). A recent study reported that *CYCLIN-DEPENDENT KINASE G2* (*AtCDKG2*) is highly expressed in flowers (Ma *et al.*, 2015), and a loss-of-function *atcdkg2* mutation caused reduced expression of the flowering repressor *FLC* under salt stress and up-regulation of flowering promotion genes such as *FT*, *AP1*, and *LEAFY* (*LFY*), leading to early flowering. Also, the *atcdkg2* mutation resulted in the up-regulation of stress-responsive genes, such as *RD29B*, *ABI2*, *MYB15*, and *P5CS1*, and thus increased salt tolerance, indicating that *AtCDKG2* normally acts as a negative regulator of salt stress response. Similarly, a study on rose (*Rosa hybrida*) flowers (Meng *et al.*, 2014) showed that rehydration (water recovery after drought stress) rapidly activates *RhMPK6* expression (a homolog of *AtMPK6*), and consequently induces the phosphorylation and accumulation of RhACS1 protein (a homolog of a key ethylene biosynthetic enzyme) in gynoecia, resulting in elevated ethylene production and downstream signaling.

Other protein modifications likely to be involved in regulation of flower development under stress include protein palmitoylation. An analysis of AtPAT10, a member of the protein *S*-acyl transferase (PAT) family implicated in palmitoylation, showed that it is crucial for reproduction and salt stress response (Zhou *et al.*, 2013). AtPAT10 regulates the *S*-acylation-dependent tonoplast association of CBL2, CBL3, and CBL6 (Feng *et al.*, 2017), a set of tonoplast calcium sensors. The two close paralogs in Arabidopsis, CBL2 and CBL3, function in both vegetative and reproductive development, with the double mutant showing impaired siliques and seeds and weak stress tolerance (R.J. Tang *et al.*, 2012).

### Metabolic enzymes and other functions

Plant cells under osmotic stresses, such as drought and salt, are known to accumulate metabolites (osmolytes) such as glycine betaine (GB) and proline to reduce dehydration (Yancey, 2005). For example, GB was found to play a role in salt tolerance during seed germination and vegetative growth (Sakamoto and Murata, 2002). GB is also important for normal plant reproductive development under drought/salt stress. Specifically, enhanced GB synthesis in transgenic Arabidopsis plants help to protect plant reproductive organs against structural defects found in the WT under salt stress and promote salt tolerance in these transgenic plants (Sulpice *et al.*, 2003).

Additionally, plant endogenous proline also plays a role in plant stress response and pollen development (Székely *et al.*, 2008; Mattioli *et al.*, 2012). The rate-limiting step of proline biosynthesis is catalyzed in Arabidopsis by enzymes encoded by closely related *AtP5CS1* and *AtP5CS2* (Strizhov *et al.*, 1997). *AtP5CS1* is detected in leaves, stems, and flowers at significant levels, while *AtP5CS2* is 3- to 5-fold lower in most of the organs. Moreover, *AtP5CS1* is more highly expressed under drought and salinity stresses, as well as after ABA treatment (Strizhov *et al.*, 1997). The differential expression in different organs of the two *AtP5CS* genes might lead to differential accumulation of proline and different degrees of stress tolerance. In addition, the loss of *AtP5CS1* and *AtP5CS2* function causes defective male gametophytes and reduced fertility, indicating that proline is required for pollen development (Mattioli *et al.*, 2012).

In addition to the above genes, other genes with distinct functions were also found to be involved in drought/salt response. A rice nucleolin gene *OsNUC1* is differentially expressed between salt-sensitive and salt-resistant rice lines under salt stress (Sripinyowanich *et al.*, 2013). Nucleolin is involved in the synthesis and maturation of ribosomes in the nucleolus. Alternative splicing results in transcripts of two sizes, *OsNUC1-S* (shorter) and *OsNUC1-L* (longer), both of which are expressed in leaves, roots, flowers, and seeds. Arabidopsis mutants specifically lacking the *AtNUC1-L* transcript but expressing the rice *OsNUC1-L* transcript exhibited a more complete revertant phenotype than the lines expressing *OsNUC1-S*. However, the *OsNUC1-S*-expressing *atnuc1-l* lines displayed a higher growth rate with longer roots and lower H_2_O_2_ levels under high salt conditions. In addition, they also showed higher levels of induced *AtP5CS1* and *AtSOS1* expression under salt stress than the WT (Sripinyowanich *et al.*, 2013).

Ion homeostasis is also related to abiotic stresses; for example, drought induces an increase in the level of free ferrous ion Fe^2+^, whereas ferritin (Fer) proteins sequester free ions to protect cells from damage. In the Arabidopsis ferritin triple mutant (*fer 1-3-4*), the elongation of stamen filaments was reduced, with a failure to position the anthers above the stigma, and stigmatic papillae rarely develop well, leading to sterility. (Ravet *et al.*, 2009; Yadav, 2010; Brini and Masmoudi, 2012; Tripathi *et al.*, 2018). Also the expression of a rose ferritin gene *RhFer1* is induced by dehydration and ABA during flower senescence. This activation under drought stress requires the function of RhABF2, an AREB/ABF family TF involved in ABA signaling, through direct binding of RhABF2 to the *RhFer1* promoter (Liu *et al.*, 2017). Subsequently, the drought-induced free ferrous ion Fe^2+^ is preferentially sequestered by RhFer1 but not transported outside of the petal cells to limit oxidative species during drought. This regulatory module with *RhABF2/RhFer1* maintains the intracellular Fe^2+^ level and enhances drought tolerance in rose flowers. Additionally, a putative rice lipid transfer protein gene *OsDIL* is expressed in the anther, primarily in response to drought, salt, cold, and ABA (Guo *et al.*, 2013). Overexpression of *OsDIL* in drought-treated rice plants led to elevated tolerance to drought stress during vegetative development and reduced anther defects at reproductive stages (Guo *et al.*, 2013). Taken together, when plants are subjected to drought/salt stresses, a variety of cellular processes could be activated, inhibited, and integrated to allow survival through the adverse environment while maximizing/protecting reproductive yield.

### Detection of drought-responsive genes using transcriptomics

In addition to the studies focusing on individual gene functions, transcriptomic studies have detected global gene expression changes during reproductive development under drought stresses in Arabidopsis and rice (Jin *et al.*, 2013; Su *et al.*, 2013; Ma *et al.*, 2014). In Arabidopsis plants treated with drought starting at the onset of flowering to the end of their life cycle (Su *et al.*, 2013), >4000 differentially expressed genes were identified in the inflorescence, including genes regulating flowering time, floral organ development, and stress responses (Su *et al.*, 2013). Different subsets of genes displayed different temporal expression patterns during the period of drought stress. A set of drought-responsive TF genes, including *AtDREB1a*, *ANAC019*, and *AREB* genes, were highly induced 3 d after drought started. Genes responsive to water deprivation and the ABA signaling pathway were enriched among those up-regulated after 5 d of drought treatment, indicating enhanced stress response; whereas genes for cell cycle, DNA unwinding, and nucleosome assembly were repressed 5 d after drought treatment, suggesting that the growth rate was lowered. Among the drought-induced genes, *AtDREB1A* and *AtMYB21* were tested functionally and found to play an important role in flower development under drought stress. As the severity of drought can be important, further studies were performed on the effects of various extents of drought (Ma *et al.*, 2014). As expected, Arabidopsis plants exhibited varying degrees of growth inhibition as the soil moisture was progressively reduced to ~65–70% (slight drought), ~45–50% (moderate drought, MD), and ~30–35% (severe drought, SD) relative amount of water. Transcriptome analyses of flowers identified >4000 genes being differentially expressed under SD and >1800 genes differentially expressed under MD. As expected, the majority of genes showed similar levels of expression under MD as compared with SD. However, a small set of genes (~220) only responded to MD, suggesting that distinct sets of genes may be responsible for growth under different amounts of water availability.

In rice, >1000 genes were differentially expressed in response to drought (Jin *et al.*, 2013). During four stages of flower development as monitored by size, most of these genes were affected in only one or two stages, suggesting that the developmental stage is a key determinant of drought response in flowers. Genes involved in anther development, cell wall formation or expansion, and various signaling pathway were uncovered, indicating interactions between reproductive development and phytohormone signaling in drought-stressed plants. Taken together, under moderate drought conditions, plants induce the genes that function in protecting it against the stresses and in ensuring reproductive success. Under severe drought, however, plants attenuate the expression of genes for reproductive development, and devote greater amounts of energy and resources to ensure survival and, when possible, facilitate modest seed production after acclimation.

## Temperature: cold/chilling and heat stresses

**Fig. 3.**
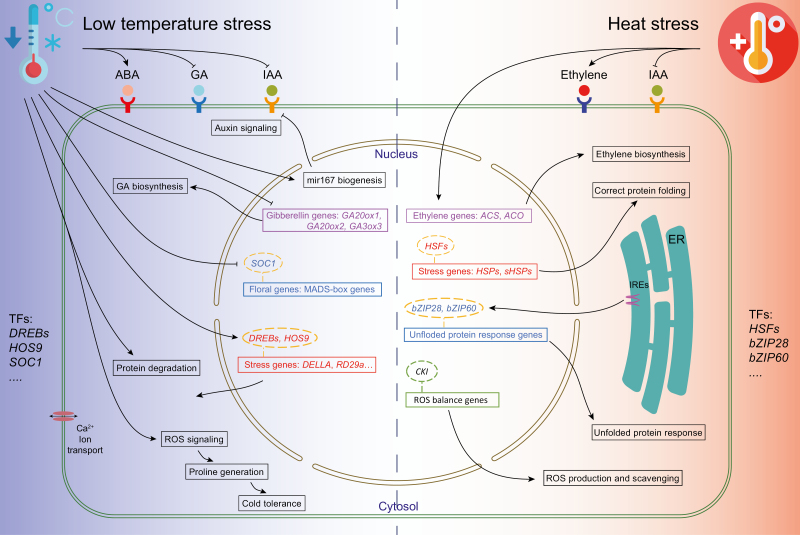
Cellular responses to temperature stresses (cold and heat) during reproductive development. Key regulators discussed in this review are shown.

Temperature is another major factor affecting the distribution and seasonal behavior of plants. Plants under cold stresses exhibit plasma membrane disintegration, cold-induced dehydration, and metabolic dysfunction (Yadav, 2010). In addition, cold-activated signaling pathways are also known to crosstalk with drought and salt stress signaling pathways (Jonak *et al.*, 1996; Yadav, 2010; Zhu, 2016). As global warming is approaching, plant species that are unable to alter flowering time in response to temperature are disappearing from their previous natural habitats, with a tendency to shift to higher altitudes and latitudes (Quint *et al.*, 2016). In Arabidopsis, heat stress induces photosynthetic acclimation, respiration, and changes in carbon balance (Quint *et al.*, 2016). Both cold and heat stresses affect plant reproductive development. Here, we review the available information and discuss how plants respond to low or high temperature stress (Fig. 3).

### Cold-responsive genes related to plant reproductive development

Different plants have adapted to growth in different temperature ranges; for Arabidopsis, 4–10 °C is considered cold (also referred to as chilling) stress, whereas near 0 °C or below is used for freezing stress (Penfield and Springthorpe, 2012; Zheng *et al.*, 2016; Cvetkovic *et al.*, 2017; Pareek *et al.*, 2017). Additionally, the temperatures for chilling and freezing stresses in rice are 0–15 °C and below 0 °C, respectively. Plants sense cold by changes in the membrane fluidity and metabolic activity. Subsequently, there are changes in cellular Ca^2+^ level, calmodulin, phytohormone and ROS signaling, and the C-REPEAT BINDING FACTOR (CBF) family TFs are activated to regulate downstream factors during reproductive development (Zinn *et al.*, 2010). Additionally, cold-induced reduction of gibberellin (GA) disrupts rice pollen development (Sakata *et al.*, 2014). The endogenous GA level and the expression of GA biosynthesis genes are repressed in rice under low temperature treatment (19 °C), while the expression levels of stress-responsive genes such as *DREB* and *DELLA* are increased. Also, in mutants defective in GA biosynthesis and response (such as a rice *ga20ox2* mutant), the application of exogenous GA was able to reduce the sensitivity to low temperature, anther defects, and male sterility caused by low temperature (Sakata *et al.*, 2014). These results suggest that the application of GA can rescue cold-induced male sterility, providing a potential remedy for agriculture to cope with global climate change. Although GA is known to impact several aspects of plant growth, development, and senescence (Hedden and Sponsel, 2015; Binenbaum *et al.*, 2018), Sakata *et al.* (2014) provided novel evidence of its role in stress response; this might form a crosstalk with ABA signaling pathways to promote stress tolerance during reproductive development.

Furthermore, other TFs are important for plant response to cold stress. One of the HOS (HIGH EXPRESSION OF OSMOTICALLY RESPONSIVE GENES) family of TF genes, *AtHOS9* (Zhu *et al.*, 2004), was identified because the *athos9* mutant is hypersensitive to freezing cold. The *athos9* mutant displays several developmental defects including slower growth rate, late flowering time, and lower trichome density. The *athos9* mutant also has increased levels of expression for some stress-responsive genes such as *AtRD29A*, but not CBF, which is a key regulator of cold response (Thomashow, 2001; Liu *et al.*, 2018). These results indicate that *AtHOS9* is important for plant growth and development, and for freezing tolerance through a CBF-independent pathway.

Another gene involved in reproductive development under cold stress is a member of the MADS-box gene family, which includes regulators of flowering time and the ABCE genes for floral organ identities (Coen and Meyerowitz, 1991; Becker and Theissen, 2003; Jack, 2004). Many MADS-box proteins share a highly conserved MADS-domain (for DNA binding and dimerization) and a moderately conserved K domain (keratin-like; important for protein interactions) (Coen and Meyerowitz, 1991; Becker and Theissen, 2003; Jack, 2004). A study was performed on the K domain of a blueberry (*Vaccinium corymbosum*) SOC1-like gene (*VcSOC1K*), for its potential role in blueberry reproduction under chilling stress (Song and Chen, 2018). The overexpression of *VcSOC1K* resulted in an increase in the number of canes, floral buds, and flower and fruit clusters under chilling treatment as compared with the WT. Transcriptome analysis identified the differential expression of 17% of the MADS-box genes and their putatively interactive genes in blueberry, which are homologous to known genes involved in flowering, phytohormones, and stress response. These results indicate that the MADS-box genes are involved in plant vegetative and reproductive development under chilling stress, possibly through interaction with other MADS-box proteins via the K domain, thereby regulating gene expression. Further studies are needed to examine the detailed floral organ structural defects, decreased seed production, and expression of the affected floral genes.

### Transcriptomic studies under cold stress

Several transcriptome studies have examined gene expression changes in floral organs under cold (Lee and Lee, 2003; Imin *et al.*, 2004; Hedhly *et al.*, 2009; Z. Tang *et al.*, 2012). Specifically, in Arabidopsis pollen (Lee and Lee, 2003), >40% of the expressed genes are possibly involved in pollen development and pollen tube growth at low temperature, including genes encoding cell wall modification enzymes. Further temporal transcriptomics indicated that the majority of transcripts were unaffected after 72 h of 0 °C chilling treatment, while genes associated with pollen tube growth and seed production were significantly reduced. However, many genes that are responsible for cold response and highly induced in leaves under chilling temperature, such as *COR* and genes for lipid transfer proteins, are only expressed at their normal level or weakly induced in the pollen. These results suggest that the reason plants are more sensitive to abiotic stress during reproductive stages is probably because of the insufficient accumulation of proteins under stress. Additionally, a study in a wheat (*Triticum aestivum*) male-sterile line investigated potential roles of small RNAs in cold response (Z. Tang *et al.*, 2012). Among 78 unique miRNA sequences, six were cold responsive. Specifically, miR167 plays roles in regulating the auxin signaling pathway, as well as possibly cold stress response and male fertility.

Moreover, a proteomic study using two-dimensional gel electrophoresis (2-DE) focused on the effect of cold stress on rice anther at the young microspore stage after 4 d of 12 °C cold treatment (Imin *et al.*, 2004) and identified 70 differentially expressed proteins. Among these proteins, 12 were newly induced compared with those at normal temperature, 47 were up-regulated, and 11 were down-regulated. Further mass spectrometry analysis revealed that seven proteins were breakdown (cleavage) products, suggesting that the cold treatment induced protein degradation. Meanwhile, the proteomic and pollen transcriptome results together demonstrate that plant stress response is a dynamic process, wherein the production and degradation of transcripts and proteins are highly regulated to help the plant survive the adversity and maximize potential productivity.

### Heat-responsive genes related to plant reproductive development

Unlike plants facing cold stress, plants under heat stress need to emit the excessive thermal energy. High temperatures, 28 °C for Arabidopsis and 32 °C for rice, affect both vegetative and reproductive development, especially male fertility (Hedhly *et al.*, 2009). Transcriptional regulation is important for heat stress response; in particular, heat shock transcription factors (HSFs) are responsible for initiating the rapid transcriptional activation of downstream genes (M. Guo *et al.*, 2016). The plant *HSF* gene family contains 21 members in Arabidopsis and 25 in rice; they are grouped into three classes A, B, and C (von Koskull-Doring *et al.*, 2007; M. Guo *et al.*, 2016). In Arabidopsis, the *AtHSFB2a* transcript has a natural long non-coding antisense RNA, *asHSFB2a* (‘as’ stands for antisense), which is only expressed after heat stress; in addition, the overexpression of *AtHSFB2a* resulted in a dramatic reduction of *asHSFB2a* expression to below the level of detection, and vice versa (Wunderlich *et al.*, 2014). In both cases, ~50% of the female gametophytes were arrested in early development, resulting in 45% ovule sterility. These results indicate that the balanced expression between *AtHSFB2a* and its natural antisense RNA regulates vegetative and gametophytic development under heat stress.

In tomato (*Solanum lycopersicum*) (Fragkostefanakis *et al.*, 2016), *SlHSFA2* (a homolog of the Arabidopsis *HSFA2* gene) acts as a co-activator of *SlHSFA1a*, and a moderate heat treatment (37.5 °C) causes the accumulation of *SlHSFA2*, thus enhancing the heat tolerance in seedlings of a subsequent severe heat stress (47.5 °C). Furthermore, repression of *SlHSFA2* expression reduced pollen viability and germination rate when heat stress was applied during early stages of pollen formation (meiosis and microspore formation) but had no effect at more advanced stages. Therefore, *SlHSFA2* is an important factor for thermotolerance of developing pollen. The results from Arabidopsis and tomato suggest that related members of the HSF family have diverged during angiosperm evolution to promote female and male reproductive development, respectively, while both still function in response to heat stress.

Another type of well-known proteins for plant responses to heat stress are heat shock proteins (HSPs) (Wang *et al.*, 2004; Volkov *et al.*, 2005; Kotak *et al.*, 2007). HSPs are molecular chaperones and are important for coping with heat-induced protein unfolding in all organisms. One class of HSPs is small HSPs (sHSPs); an example of an sHSP is the Arabidopsis BOBBER1 (AtBOB1) protein, which also contains a NudC domain (Nuclear Distribution C, important for protein folding and interaction as a chaperone) (Jurkuta *et al.*, 2009). An *atbob1* mutant is deficient in heat tolerance and exhibits defects in flowers and inflorescence meristems, suggesting its role in both development and thermotolerance (Perez *et al.*, 2009).

Heat stresses cause abnormal protein folding, and trigger a process called the unfolded protein response (UPR) (Ruberti *et al.*, 2015; Bao and Howell, 2017). Genes related to the UPR have also been found to play roles in reproduction. In particular, the *IRE1* (*INOSITOL REQUIRING ENZYME 1*, encoding an RNase) gene acts downstream of UPR and is important for proper splicing of the *bZIP60* mRNA encoding a transcriptional regulator (Deng *et al.*, 2011; Nagashima *et al.*, 2011). The *ire1a-2 ire1b-4* double mutant is fertile at room temperature but male sterile at 27.5 °C, with defects in tapetum morphology (Deng *et al.*, 2016). Further analysis indicated that the RNase activity of AtIRE1 was required for promoting male fertility under heat stress (Deng *et al.*, 2016). Further studies are needed to uncover the mechanisms by which these transcriptional regulators improve reproduction under heat stresses.

Another study in cotton (*Gossypium hirsutum*) revealed that GhCKI (casein kinase I) plays a role in male reproduction under high temperature (Min *et al.*, 2013). The mammalian CKI homologs are involved in inhibition of glycogen synthesis and regulation of apoptosis (Peters *et al.*, 1999; Price, 2006). *GhCKI* is induced after heat treatment and activates ABA accumulation (Min *et al.*, 2013). In addition, overexpression of *GhCKI* in Arabidopsis caused disruption of ROS balance and failure in tapetal programmed cell death, eventually leading to anther abortion, indicating that *GhCKI* might be used in genetic engineering to promote male fertility under heat stress.

Phytohormone signaling is also involved in plant heat response during reproductive development. Ethylene biosynthesis and signaling in floral and fruit tissues under high temperature were analyzed using pea (*Pisum sativum*) (Savada *et al.*, 2017). The mRNAs of two key genes of ethylene biosynthesis, *PsACS* and *PsACO*, were highly abundant in ovaries and pedicels, and further elevated upon heat stress. The expression level of *PsACS* and *PsACO* and the ethylene level were further detected in heat-treated floral organs, including ovary, pedicel, anther, stigma, and petal. The results demonstrated that in contrast to untreated plants, heat-treated plants facilitate the senescence of unpollinated ovary, and inhibit the development of petal and the growth of the pollen tube by regulating *PsACS* and *PsACO* expression and ethylene levels, allowing the allocation of limited resources for seed development and maturation from the fertilized ovules. This suggests that ethylene biosynthesis is involved in resource allocation during reproductive development under heat stress.

Finally, siRNAs are also involved in heat stress response (Ito *et al.*, 2011). A *copia*-type retrotransposon *ONSEN* is induced by heat treatment in siRNA biogenesis-deficient Arabidopsis plants. In the progeny of heat-stressed siRNA biogenesis-deficient plants, a high frequency of new *ONSEN* insertions was observed; these insertions were subsequently demonstrated to have occurred during flower development and before gametogenesis. These findings suggest that *ONSEN* insertions may confer heat responsiveness to nearby genes, leading to the activation of stress-responsive gene regulation during reproductive development.

### Transcriptome studies and other large-scale analysis under heat stress

Transcriptome studies have been performed in Arabidopsis, wheat, and cotton (Chauhan *et al.*, 2011; Baron *et al.*, 2012; Min *et al.*, 2014; Zhang *et al.*, 2017). A study in Arabidopsis examined expression responses of 17 genes involved in ABA metabolism (Baron *et al.*, 2012), including biosynthesis, catabolism, and transport, in different tissues under heat stresses. The results demonstrated that a subset of genes responsible for ABA biosynthesis, hydrolysis, and transportation are differentially regulated in an organ-specific pattern; in addition, both cold and heat stresses negatively affected reproductive organ development. For example, a key enzyme involved in ABA biosynthesis, *AtNCED3*, is significantly reduced in leaves under either cold or heat stress, but significantly induced in inflorescence meristem by cold and heat stress.

Moreover, a recent transcriptomic study under heat stress uncovered 484 up-regulated and 373 down-regulated genes in the Arabidopsis flower (Zhang *et al.*, 2017). As previously mentioned, genes related to the endoplasmic reticulum (ER) UPR, protein ubiquitination, and protein folding were enriched. In Arabidopsis, bZIP28 and bZIP60 are the major transcriptional regulators of the UPR, and are induced under heat stress (Deng *et al.*, 2011; Song *et al.*, 2015). *Atbzip28 bzip60* double mutant plants exhibited reduced silique length as well as fertility, and a comparison of the transcriptome between the WT and *Atbzip28 bzip60* double mutant identified 521 differentially expressed genes under heat stress in reproductive tissues, suggesting protective roles of the UPR for maintaining fertility upon heat stress. (Zhang *et al.*, 2017).

Another transcriptomic study in wheat explored heat-responsive genes specifically induced at different vegetative and reproductive stages (Chauhan *et al.*, 2011). Among 127 genes highly induced in the flower were genes for TFs such as a C3HC4-type zinc finger protein and the HSP TaHSP82, as well as other proteins including a 14-3-3 protein, chitinase, and calmodulin (*TaCAM3-1).* Similarly, comparative transcriptomics was performed in cotton developing anther under heat stress (Min *et al.*, 2014), identifying 4599 differentially expressed genes. The functional categories identified among the differentially expressed genes included those for epigenetic modifications, carbohydrate metabolism, and phytohormone signaling. Specifically, *GhCKI* (also mentioned in the previous subsection) was identified by the transcriptome and displayed induction by heat stress, as well as the *GhPIF* (*PHYTOCHROME-INTERACTING FACTOR*) gene, which is involved in sugar and auxin (indole-3-acetic acid, IAA) signaling. In short, multiple processes, such as transcriptional regulation, protein conformational changes, ion transportation, and phytohormone signaling, are involved in response to heat stress during anther development.

In addition to transcriptomic studies, a genome-wide association study (GWAS) has been conducted to investigate Arabidopsis flower and seed development under heat stress (Bac-Molenaar *et al.*, 2015; Luria *et al.*, 2019). The effect of heat stress on flower and fruit development is highly similar to what was reported under drought stress (Su *et al.*, 2013). These authors found that two developmental stages are particularly sensitive to high temperature: one before anthesis near the time of male and female meiosis, and one after anthesis during fertilization and early embryo development. They further identified four quantitative trait loci (QTLs) that are strongly associated with heat response and are developmental stage specific. These results are in agreement with previous conclusions (Kim *et al.*, 2001; Warner and Erwin, 2005) that the regulation of heat tolerance is likely to be dependent on stages.

## Oxidative stress

ROS, including superoxide (O2· ^–^), hydrogen peroxide (H_2_O_2_), and hydroxyl radicals (OH·), are present in plants even under non-stressful growth conditions. Oxidative stress arises from the imbalance between generation and elimination of ROS and could eventually lead to cell death (Scandalios, 2002). Oxidative stress is often a component of responses to various abiotic stresses such as drought, salinity, high and low temperature, and heavy metal stress. Nevertheless, oxidative stress also has its own signaling mechanisms independent from the signaling pathways triggered by the other stresses mentioned above (Hasanuzzaman *et al.*, 2013; Krishnamurthy and Rathinasabapathi, 2013). To date, there are only a few reports on the effect of oxidative stress on plant reproductive development. Oxidoreductases, by definition, play important roles during flower development in response to the oxidative processes (Xing *et al.*, 2005; Wang *et al.*, 2009; Blee *et al.*, 2014; Dai *et al.*, 2019). For example, glutaredoxins (GRXs), a kind of oxidoreductase, displayed a conserved function in response to oxidative stress during flower development, as supported by the crucial functions of two Arabidopsis CC-type GRX genes, *AtROXY1* and *AtROXY2*, in petal and anther initiation and differentiation (Xing *et al.*, 2005) and similar functions of the two rice homologs *OsROXY1* and *OsROXY2* (Wang *et al.*, 2009). They display the same expression pattern in floral organs, and overexpression lines exhibit the same flower developmental defects and increased H_2_O_2_ accumulation. Another Arabidopsis protein AtADR (ANTHER DEHISCENCE REPRESSOR) (Dai *et al.*, 2019) is *N*-myristoylated and targeted to peroxisomes during early flower development. Ectopic expression of *AtADR* in transgenic Arabidopsis plants resulted in male sterility due to anther indehiscence probably mediated by reduced ROS accumulation and repressed expression of *AtNST1* and *AtNST2*, which are required for anther dehiscence.

ROS imbalance can also be regulated through changes in ion level, thus ion transportation and homeostasis are possible processes involved in responses to oxidative stress. The Arabidopsis membrane-spanning protein AtPIC1 (PERMEASE IN CHLOROPLASTS 1) mediates iron transport across the chloroplast inner membrane, helps ferritins store free iron, and reduces the cellular ROS level (Duy *et al.*, 2007). The overexpression of *AtPIC1* leads to iron overload in chloroplasts and leaf chlorosis (oxidative-stressed phenotype), as well as severely defective flower and seed development (Duy *et al.*, 2011). Another Arabidopsis TF gene *AtSPL7* (*SQUAMOSA PROMOTER-BINDING PROTEIN-LIKE7*) is responsive to copper insufficiency (Garcia-Molina *et al.*, 2014). AtSPL7 interacts directly with Arabidopsis AtKIN17 in response to copper deficiency, and the *atspl7 atkin17* double mutant displays an enhanced copper-dependent oxidative stress, floral bud abortion, and pollen infertility (Garcia-Molina *et al.*, 2014). These results support the idea that ion homeostasis in plant cells is important for response to oxidative stress and flower and fruit development.

## Light stress and radiation

Light affects almost every aspect of plant development, from germination to flowering. Although plants respond to a broad spectrum of light, ranging from UV-B to far-red light, light overdose can be harmful to plant growth and development (Chory *et al.*, 1996; Fankhauser and Chory, 1997). Also, X-rays are harmful to all organisms, including plants, by, for example, causing genomic DNA breakage. The Arabidopsis recombination endonuclease gene *AtMRE11* plays important roles in DNA damage sensing and repair (D’Amours and Jackson, 2002; Puizina *et al.*, 2004; Stracker and Petrini, 2011). An Arabidopsis histone acetyltransferase, AtTAF1, which also associates with the TATA-binding protein, interacts with AtMRE11 and is important for male gametophyte development (Waterworth *et al.*, 2015). Heterozygous *AtTAF1*^*+/–*^ plants display defects in pollen tube development, reduced fertility, and hypersensitivity to X-rays, suggesting that *AtTAF1* acts together with *AtMRE11* to contribute to sensing and repair of DNA damage and also to male fertility.

In addition, it was found that UV-C light activates the flowering transition in Arabidopsis through salicylic acid (SA) (Martinez *et al.*, 2004). Also in non-stressed plants, SA acts as a regulator of flowering by activating pathways independent of known flowering regulators [*CONSTANS* (*CO*), *FCA*, or *FLC*] to regulate flower development. For plant response to UV-B light, a comparative transcriptomic analysis between Arabidopsis and *Physcomitrella patens* reveals both genes in conserved pathways and those that are likely to represent distinct processes (Wolf *et al.*, 2010). Among the conserved genes, some are annotated to encode calcium-binding protein, hydrolase, oxidoreductase, and proteins similar to harpin-induced proteins, which are also associated with oxidative stress.

## Future perspectives

Plants are highly sensitive to environmental stress during reproductive development, and respond dramatically to allow resource re-allocation and maximize reproductive success under unfavorable growth conditions. Although it is important to understand how plants respond to different abiotic stresses during reproductive development, this is still a clearly underinvestigated area. In particular, there have been few detailed studies, even in model plants, to document specific morphological and molecular changes at different stages, and for different aspects, of the complex reproductive process, in response to various conditions. In addition, great efforts are needed to understand the molecular mechanisms underlying the changes in specific reproductive processes during the acclimation to abiotic stresses. Furthermore, experiments on interactions among regulatory genes and signaling pathways induced by different stresses promise to present an integrative portrait of multiple environmental conditions that affect reproductive cellular and molecular processes. Such analyses in the near future will lead to better understanding of various aspects of reproductive development under stress, and uncover the molecular strategies that facilitate optimal reproduction that have contributed to the evolutionary successes of flowering plants.

Moreover, the homologs of many genes identified in one plant have not been characterized in most other plants, including crops, for their stress-induced expression and potential role in stress response during reproduction. Because stress response is a highly adaptive process, many genes probably have experienced functional divergence during angiosperm evolution, making comparative studies between model plants and crops both challenging and necessary. In particular, gene duplication has occurred frequently in the angiosperm history, and differential functional specialization among duplicated genes, such as between leaves and flowers, might both occur widely and vary in specific aspects among species. Such divergence might have contributed to the adaptation of different plant groups to various environments and to their success in different ecosystems. Further research will be likely to provide valuable information that will contribute improvements in agriculture and protection of ecosystems.
